# Mating and Sexual Selection in Empidine Dance Flies (Empididae)

**DOI:** 10.3390/insects13090839

**Published:** 2022-09-15

**Authors:** Rosalind L. Murray, Darryl T. Gwynne, Luc F. Bussière

**Affiliations:** 1 Biology Department, University of Toronto Mississauga, Mississauga, ON L5L 1C6, Canada; 2 Department of Ecology and Evolutionary Biology, University of Toronto, Toronto, ON M5S 3B2, Canada; 3 Department of Biological and Environmental Sciences & Gothenburg Global Biodiversity Centre, University of Gothenburg, 405 30 Göteborg, Sweden

**Keywords:** mating system, female ornamentation, nuptial gift, sexual dimorphism

## Abstract

**Simple Summary:**

In many species of *Empis*, *Rhamphomyia* and *Hilara* dance flies, females rather than males display ornaments prior to mating. These ornaments appear to have evolved by Darwinian sexual selection in which females compete for access to choosy males that supply courtship (nuptial) gifts, which in many species appear to be the main or only source of dietary protein for adult females. We review the diversity of mating in this group of flies, including the different types of ornaments and aspects of behaviour and morphology thought to influence sexual selection on each sex, including nuptial gifts, and the sex ratios of aerial swarms where pairing takes place.

**Abstract:**

Species whose behaviour or morphology diverges from typical patterns can provide unique insights on the evolutionary forces that promote diversity. Darwin recognised that while elaborate sexually selected traits mostly occurred among males, in a few species females possess such traits. Some species from the subfamily Empidinae (Diptera: Empididae) are among the animals that are often invoked to illustrate female ornaments. Empidines include taxa that exhibit varying levels of female ornament expression; some species possess multiple, elaborate female-specific ornaments while others have fewer and more modest adornments, and many species are altogether lacking discernible sexual ornamentation. This continuous variation in display traits in the Empidinae provides unique opportunities to explore the causes and consequences of sexually selected ornament expression. Here, we review the literature on sexual selection and mating systems in these flies and synthesise the evidence for various evolutionary forces that could conceivably create this impressive morphological and behavioural diversity, despite evolutionary constraints on female ornament exaggeration that help to explain its general rarity among animals. We also suggest some aspects of diversity that remain relatively unexplored or poorly understood, and close by offering suggestions for future research progress in the evolutionary ecology of mating behaviour among empidine flies.

## 1. Introduction

**Figure 1:**
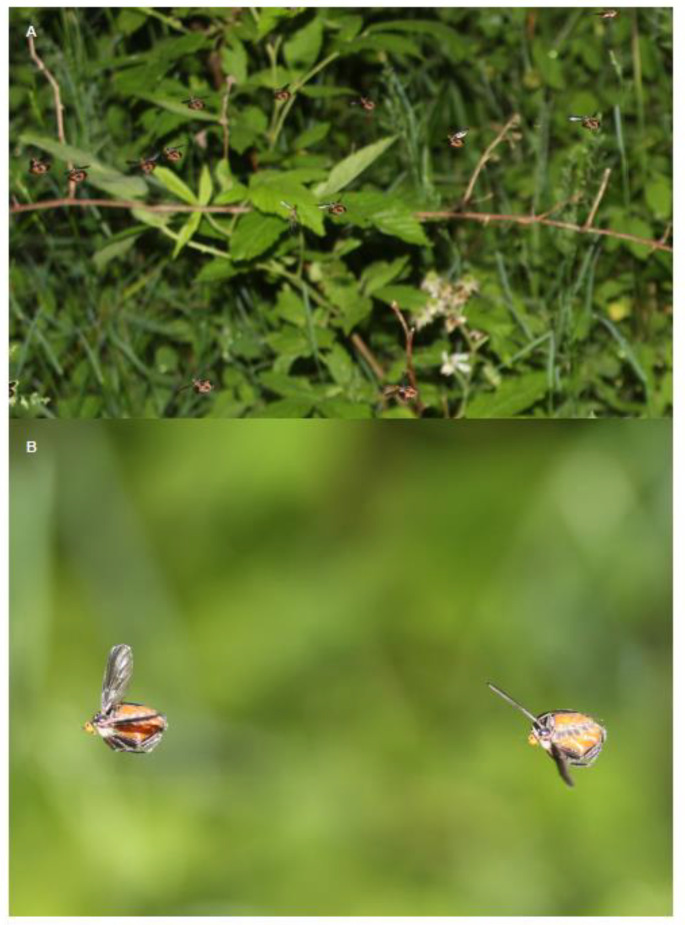
*Rhamphomyia longicauda* mating swarm. (**A**) A female-biased mating swarm showing 13 females displaying multiple ornaments and one male (centre) carrying a nuptial gift. (**B**) Two female *R. longicauda* individuals in a mating swarm displaying their ornaments—extended abdominal sacs and three pairs of legs with extensive pinnate leg scales. Reprinted with permission from John Alcock.

Females of many species of empidine flies sport sex-specific ornaments [1], some of which have a degree of elaboration that rivals both the few other well-known examples of female finery among vertebrates—e.g., flashy colouration in some pipefishes—and more commonly the gaudy traits of males such as elaborate plumage in many birds. When viewing the swarming females of *Rhamphomyia longicauda* on a June evening in southern Ontario, they can be seen moving slowly back and forth displaying two types of ornaments to (non-ornamented) males bearing prey gifts that fly up from below (Figure 1). Females display grossly enlarged (air-filled) abdominal sacs and rows of tibial scales along their legs [2].

Such ornaments were at first a problem for Darwin’s theory of natural selection (from his letters to Asa Gray [3], “…a peacock’s tail makes me sick” [4]) but inspired him to propose sexual selection, generally defined as selection for traits that evolved during competition to fertilise gametes (almost always among males) and for matings (or the best mates) per se [5]). Recent re-definitions of sexual selection [6] narrowed to just competition for gametes exclude virtually all female versions of the ornaments that puzzled Darwin because most female traits have evolved in competition for access to the goods and services that come with matings (for example, pipefish male care and dance fly prey gifts). For this review, we use the broader definition of sexual selection that includes selection on traits improving access to mates (including access to material benefits, such as nuptial gifts and to gametes) [7]. 

Virtually all information on empidoid mating comes from species of *Empis*, *Hilara* and *Rhamphomyia*, three closely related empid genera that exhibit conspicuous swarming, female ornaments and the mating and nuptial-feeding behaviours that take place within swarms. Table 1 features a summary of some species whose behaviour or morphology has been studied in some detail, alongside references. In most species, females obtain necessary protein for egg development from exogenous (sensu [8]) nuptial gifts of captured prey and, in a few cases, endogenous male glandular products [1,9,10]).

For empids, swarming behaviour and nuptial feeding have been subject to evolutionary studies. Kessel [11] suggested an evolved behavioural sequence in mate feeding beginning with species in which the sexes fed independently, through stages where freshly captured prey gifts are handed over to females. The next stages envisioned by Kessel [11] concern “empty” gifts of presumed low nutritional value. These are the “balloon flies” where males secrete endogenous balloon gifts of silk or foam [1]. In some species, balloons are wrapped around parts of dried prey or plants. In the final stage, gifts are silken balloons with no other components. 

Two decades after Kessel’s paper [11], with a renewed interest in Darwinian sexual selection and sex differences (especially theory on how relative parental investment by the sexes controls sex differences [12,13]), empid nuptial feeding was one of Thornhill’s [14] examples of male parental investment when he suggested that males of some insect species feed their mates and thus offset the disparity in relative parental investment of the sexes in zygotes. One hypothesis (Thornhill [14]) proposed was that female preferences for greater male investment in nuptial gifts could lead to the evolution of “all types of male investment patterns in insects”. 

Later, a second hypothesis [15,16,17] focused on the consequences of nutritious valuable male gifts offsetting the disparity in relative parental investment (sexual difference theory; [12,13]). When males invest in goods and services valuable to females, typical sex differences in competition, courtship and mate choice are expected to be less distinct (e.g., both sexes choose mates or possess sexually selected structures). In cases when the male contributions are sufficiently important to female fitness, there will be a reversal in the mating roles: females compete for mates (gifts) and males choose mates. The second hypothesis has been the focus of much recent research on empid mating. 

Here, we review the evolutionary significance of both swarming behaviour and the transfer of nuptial gifts in Empidoidea, addressing the fitness benefits and possible costs of these behaviours. We discuss the diversity of empidoid male gifts and swarming behaviour (including variation in swarm sex ratios as an important influence on sexual selection) then review the evolutionary consequences of gifts mainly in the context of hypotheses prompted by Thornhill [14,15]. We highlight how sexual competition among females for multiple nutritious gifts (matings) can produce sexually selected female ornaments and lead to post-copulatory sexual selection among males First, we consider hypotheses for the evolution of empid gifts. Did nutritious gifts evolve via female preferences or other means (e.g., to prevent sexual cannibalism)? Did non-nutritious balloon gifts also evolve by female choice, as, for example, “displays of male fitness” [1] or are they coercive or cheating male mating tactics? Finally, we address conflict in the fitness interests of males and females, particularly the possibility that ornamented females deceive males in their competition to acquire nuptial gifts.

## 2. Nuptial Gifts

Nuptial gifts offered by males are known from species in four genera of empidoids (although the mating habits of species in most genera are unknown) [18]. *Clinocera* and *Wiedemannia* species (Clinocerinae), in a near clade to the well-studied empidinae clade containing *Hilara*, *Empis* and *Rhamphomyia* [19], form large aggregations on riparian rock faces but do not use gifts in mating [20,21]. However, a basal empidoid and outgroup to all these genera [19], *Alavesia* (with both extant species and a species—with its gift—found in 100 myo Myenmar amber), uses “frothy” balloon gifts, but apparently does not swarm [18]. There are two conclusions from these studies: (1) gifts have probably evolved independently in *Alavesia* and in empids; and (2) the presence of balloon gifts in a basal empidoid is not consistent with Kessel’s [11] hypothesis about an evolved sequence of empidoid gift types [18] (although the appropriate test would take advantage of the diversity of gift types within the three empidine genera).

Empid gifts vary greatly [1]. Many species use newly captured prey, e.g., in *Rhamphomyia* [22,23,24] and some *Empis* and *Hilara* species [25,26]. Others (some *Hilara* and *Rhamphomyia*) use plant parts alone (typically in species where most males offer fresh prey gifts but “cheats” offer low-quality plant and dried insect parts; see below for more on sexual conflict). This has been observed in some *Empis* and *Rhamphomyia* [23,27]. 

Other species use endogenous balloons as gifts, with a few being inedible and/or not eaten by the female [1,28]. A few *Hilara* species lightly wrap willow seeds in silk [1]. Some *Hilara* and *Empis* use empty balloons consisting of inedible silk or bubbles of foam coated with filaments [1]. In some flies, filamentous balloons have embedded prey (e.g., [11,29]). Finally, *Empis trigramma* males ejaculate a “liquid gift” into the female which she then discharges and eats [27]. In a few *Rhamphomyia*, *Empis* and *Hilara* species, there is no discernible gift [1,30,31]. These apparent evolutionary losses of gift-giving appear to be a result of shifts to mating on the ground or to nectar feeding [1]. For example, the nectar-feeding *Rhamphomyia magellensis* has lost both gift-giving and swarming; courtship and mating occur on plants [32]. 

Several hypotheses have been suggested for the adaptive significance of mate feeding by male empidoids. Melander [33] and Kessel [11] argued that the gift functioned to avoid sexual cannibalism by distracting the mating female with a meal. Downes [34] viewed this as unlikely because most empid females do not appear to hunt any prey and there are no observations of females attacking males. Thornhill [14] stated that the hypothesis lacks behavioural support and that males as prey may be accidental (e.g., in some species, males capture conspecific males as nuptial prey [27]). Svensson and Petersson [26] also dismissed this hypothesis as they did not observe sexual cannibalism in their long-term study of *Empis borealis*. 

In his study of several *Rhamphomyia* species, Downes [10] favoured the hypothesis that nuptial prey are important to female egg development but did not address male fitness. In contrast, Alcock [25] stated that by feeding their mates, *Empis borealis* males give “their gametes a boost” and may also contribute to female somatic maintenance so that “the success of the male’s genetic contribution is intimately tied to the survival and reproduction of each of his mates”. Based on his studies showing that females of *Hylobittacus* hangingflies (Mecoptera) favour males that handed over large nuptial prey, such as by increasing copulation duration [35], thus maximising the number of sperm transferred, Thornhill [14] pointed to female mate choice as driving the evolution of mate feeding in all insects, including empidids. By experimentally manipulating *Rhamphomyia sulcata* prey size, LeBas and Hockham [23] tested part of this hypothesis by showing that larger food gifts resulted in a longer copulation duration. Svensson and Petersson [26] also showed that male *E. borealis* exert mate choice and were the first to suggest a reversal in the mating/courtship roles for a dance fly with large nutritious prey gifts.

In contrast to dance flies with nutritious gifts are those in which males provide “empty” gifts. These include both species with gifts not eaten by the female, and species where males typically offer fresh prey but where some males attempt to get matings using items such as dried insect parts or fluffy seeds. 

Inedible balloon gifts have been viewed as ritualised behaviour or necessary “sign stimuli” preceding copulation [9,11]. Putting this in male fitness terms, Cumming [1] concluded that “ritualised” balloon gifts were “displays of male fitness”, reflecting Thornhill and Alcock’s [36] suggestion that balloons were indicators of male condition, honestly advertising his ability to forage. This hypothesis predicts female preferences for larger balloons, and was refuted using *Empis snoddyi*, a species with an inedible gift balloon. Sadowski, Moore, and Brodie [28] concluded that sexual selection favoured increased male body size but that males with intermediate-sized balloons obtained higher mating success. They concluded that gift size may still signal male quality to potential mates, but that the net consequence of female preference for large balloons and their hindering effects on flight might be stabilising selection. 

An alternative to the hypothesis that non-nutritious gifts are cooperative signals (non-antagonistic signals that benefit both signaler and receiver) of male quality is that they represent attempts by males to deceive females (see section on sexual conflict below). This possibility is supported by instances of gift polymorphism, where many matings involve fresh prey but others use virtually inedible dried insect or plants parts, which has been observed in a number of empids. For two *Empis* species, Preston-Mafham [27] showed that such males achieved shorter copulations than males with fresh gifts, and suggested that dried prey were carried by males using an alternative “cheat” mating strategy. LeBas and Hockham [23] replaced the gift of male *Rhamphomyia sulcata* with four types of experimental prey. Copulation duration was longest with large fresh gifts and both small and large dry “token” gifts were equivalent to a small fresh gift, leading to the conclusion that males can cheat and obtain some reproductive success by using gifts of low nutritional value.

## 3. Mating Swarms

**Figure 2:**
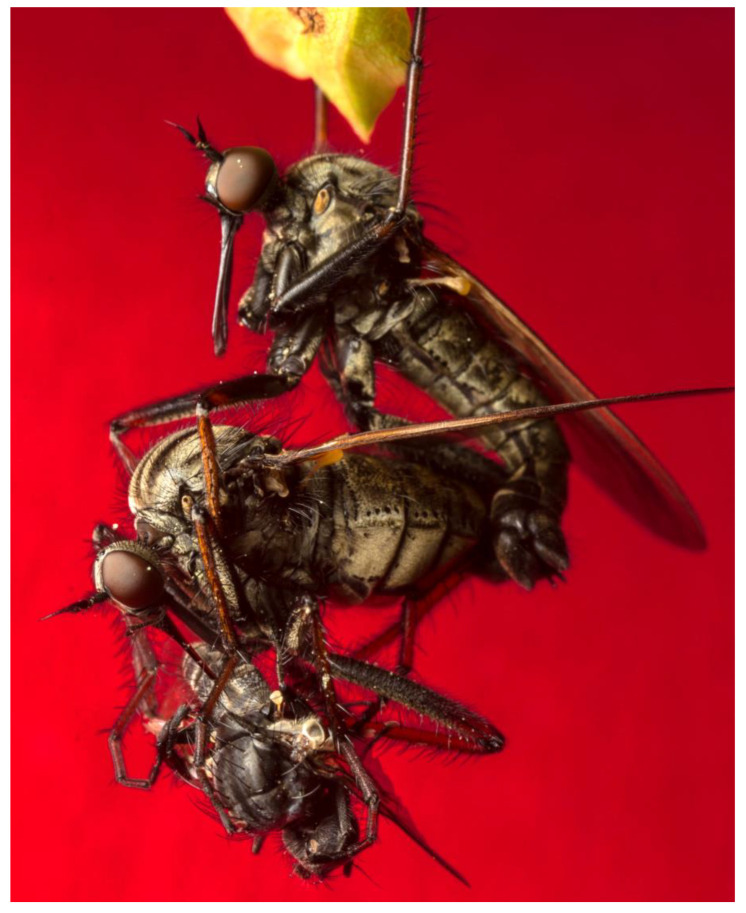
Mating *Empis tessellata*; the pair lands on a substrate and the male supports the feeding female during copulation. **Top**: male; centre: female; **bottom**: nuptial gift. Note that the nuptial gift here is another empidine, *Rhamphomyia crassisrostris*. Reprinted with permission from Tom Houslay.

At the centre of the courtship and mating of the empidines is the mating swarm, in which courtship, coupling, gift exchange (when present), and sometimes also sperm transfer can take place. Here, we describe the general features of mating swarms, while remaining cognizant of: (1) diversity, both within and among species, in the size, density, composition, and activities occurring within swarms, and (2) the fact that our knowledge is limited to detailed descriptions from a small number of taxa. For most species, swarms have never been described in detail, perhaps because swarming is altogether absent. However, even in species where swarms are known to occur, they may be relatively inaccessible to human observers or so diffuse (with single individuals dancing apparently alone) as to be practically undetectable. The typical swarm of dance flies (Empidinae) is aerial and involves flies remaining on the wing for long periods while courtship and sexual competition take place ([37]; see also Figure 1 in [2]). In other cases, aggregations involve perching on vegetation except for irregular bursts of displays of mating flight upon the arrival of a prospective mate (e.g., *E. barbatoides* and *E. poplitea* [25]; *E. tessellata*, Figure 2). In still other cases, swarming appears to be a strictly terrestrial affair (e.g., *E. trigramma* [30]). Following pairing, mating can occur exclusively on the wing, often involving a prolonged period of circling flight near the mating swarm (e.g., *R. sociabilis* [37]; *R. longicauda*, [2]), or can involve perching on vegetation during which time feeding and sperm transfer take place (e.g., *E. barbatoides* and *E. poplitea* [25]).

Aerial mating swarms typically involve “quasi-stationary flight over a landmark, often undertaken by many insects together, and during which mating takes place” [38]. It is challenging to describe what a swarm landmark is in a universally applicable way, but some features of the landscape reliably elicit swarming behaviour. For example, in his description of the behaviour of several arctic dance flies, Downes [10] was able to lay down black sheets as landmarks that soon became new swarming locations. *R. fumosa* is associated with tall ferns [39], while *E. snoddyi* swarms at conspicuous features, such as a bush, rock, or tree stump [28]. *R. longicauda* swarms under gaps within the canopy that facilitate the assessment from below of female silhouettes [2]. For many species, however, the exact nature of swarm landmarks remains mysterious, and appears less clearly related to notable habitat characteristics (e.g., in *R. sociabilis* [37] and *R. marginata* [40]). Nevertheless, swarms often appear reliably in the same location day after day, year after year (e.g., *E. opaca* and *E. tessellata* [27]), which indicates some consensus among the flies despite uncertainty among scientists. In our own observations, we have occasionally found that swarm sites became more or less fashionable depending on the presence or position of surrounding vegetation, for example, following storm damage or gardening activity. In some cases, swarms can appear and dissipate depending on the absence (*E. trigramma*) or presence (*E. tessellata*) of the sun in the sky, or the prevailing direction of wind [27]. Most authors have commented that swarms of particular species tend to occur within a constrained daily rhythm, e.g., mainly in the morning (*E. snoddyi* [28]), late afternoon (*Rhamphomyia* sp. [41]), or at dusk and dawn (*R. longicauda* [42]). There may well be a further social dimension to swarming landmarks, in that a key marker includes the presence of swarming conspecifics, whether those are detected visually or via acoustic cues of swarming flight. 

The adaptive significance of social swarms (as opposed to displays performed in isolation) have not been systematically examined for dance flies. One possibility is that conspicuous displays bring about substantial predation risk, which is ameliorated by the predator confusion that swarming can cause [43]. Another possibility is that aggregations of displaying individuals are more attractive to the opposite sex (presumably because of the increased efficiency of mate choice in an aggregated setting), which more than outweighs the risk of losing to competitors. For nuptial feeding insects such as dance flies, such mate choice involves not only contrasts of the suitors but also of their gift offerings [36]. However, displaying in a swarm also heightens contests for opposite-sex partners that approach while assessing potential mates. It may be that some positions within mating swarms provide special advantage, and are competed over in a similar way to the central places in male grouse leks [44] or the bottom of the swarm in males of a bibionid fly [45]. Indeed, there is some evidence that female flies low in swarms of *R. longicauda* (Figure 1) are larger than those on the periphery [46], and that females in the centre of the swarm are more attractive [47]. Contests within swarm, whether for positional or other advantages, will be especially important when one sex is rare relative to the other. As a consequence, the factor that controls the relative numbers of males and females in mating swarms—the operational sex ratio [48]—is likely to play a large role in the nature of empidine mating systems.

## 4. Operational Sex Ratio Variation and Its Causes

One of the most notable sources of variation among dance fly swarms concerns the sex ratio within mating swarms. Swarms vary substantially from highly male-biased to highly female-biased, and demonstrate apparently continuous variation in these ratios, both among species and sometimes within them (e.g., *E. barbatoides* [25]; *E. tessellata* [27]). These differences in sex ratio probably play a central role in the behavioural diversity for which dance flies are famous, because sexual selection intensities themselves derive in large part from the intensities of contests within the sexes for access to opposite sex partners, their gifts, and their gametes [49].

The causes of swarm sex ratio variation are not clear, but some of the within-species variation is undoubtedly due to stochastic changes in the number of available mates due to chance fluctuations in attendance. While these variations can help explain individual variation, they are less useful for explaining differences in morphology and mating systems among taxa, which probably instead relate to variation in several factors: the overall adult sex ratio (ASR), which could itself depend on sex biases in mortality including sex-ratio-distorting cellular endosymbionts, the ability of males to participate in swarming (eligibility, which may depend on the procurement or production of a nuptial gift), the sexual receptivity of females, which may in turn depend on the nutritional importance of nuptial gifts, and the costs and benefits of swarming itself, which may depend on energetic reserves required for sustained flight. 

The adult sex ratio could affect swarm attendance because of sex differences in intrinsic or extrinsic demographic patterns. For example, if females emerge as adults earlier than males do, one might expect early swarms to be relatively female-biased compared to swarms later in the season. Conversely, if one sex suffers heightened mortality, such as when finding and acquiring nuptial gifts is a risky endeavour for males, or swarming females suffer greater spider predation [50], one might expect this heightened predation risk to cause late-season shifts in sex ratios. To our knowledge, there have not yet been any studies of sex differences in demography, and how they co-vary with swarm sex ratios, so any future work would be a welcome contribution.

Sex-ratio-distorting symbionts are relatively prevalent in arthropods, and known to sometimes cause skewed sex ratios that affect mating systems [51]. Biases in adult sex ratio are challenging to assess, because even if sampling methods detect larger numbers of one sex, it is unclear if behavioural sex differences lead to differential capture notwithstanding even sex ratios. Murray and co-authors [52] quantified adult sex ratios using two methods (vegetation sweep netting and Malaise trapping) and compared these to swarm sex ratios in up to 20 species (note that not all species could be observed swarming, and the habitat sampling methods did not provide adult sex ratios for all species in which swarms were observed). While there were significant departures from unity sex ratios outside the swarm for a few species (*E. tessellata* and *R. longipes* for vegetation samples, and *E. nigripes*, *R. dentipes*, *R. longipes*, and *R. tibiella* in Malaise samples), these departures were not always consistent across sampling regimes (e.g., for *R. longipes*, vegetation samples were consistently female-biased, while Malaise samples were consistently male-biased). Moreover, the skews in adult sex ratios did not reliably co-vary with swarm sex ratios, and there was no evidence that symbionts were strongly associated with biased adult or swarm sex ratios [52]. Collectively, these findings provide no support for the hypothesis that sex-ratio-distorting symbionts are causing skewed adult sex ratios that affect mating systems in dance flies. 

Variation among males in procuring or producing nuptial gifts could also account for variation in swarm sex ratios. For example, when gifts are particularly difficult to obtain or produce, but such gifts are a necessary prerequisite to successful pair formation, we expect fewer males will be qualified to participate in mating (sensu [53]). Males in some taxa are known to attempt gift theft from other males [27], or to “recycle” nuptial gifts, using each gift for more than one mating attempt and thereby alleviating the need to hunt or regenerate gifts between matings. The extent to which such processes might favour morphological prey acquisition traits is unclear, but possible targets are traits involving prey detection (e.g., eyes [10,36]) or prey capture (e.g., leg spines [54]). Such pressure seems to have led to several instances of male deception, as well, in which less costly or more easily obtained deceptive gifts are offered to females ([23,27]; see section on sexual conflict below). Variation in gift size or quality may also affect mate choice, but it will likely have a larger effect on copulation duration than on pre-mating choice (see section of post-copulatory sexual selection below).

Variation in female receptivity has been suggested as a primary source of comparative variation in mating systems. Cumming [1] posited that among-species difference in the reliance of females on adult dietary protein might explain a large fraction of the variation in swarm sex ratios, with relatively anautogenous females (those relying more heavily on dietary protein provided by mates for ovigenesis) becoming more highly polyandrous. This hypothesis has not been systematically tested, but Hunter and Bussière [55] provided some support for it by comparing ovarian development in one ornamented (*E. aestiva*) and one unornamented (*R. crassirostris*) species; as predicted by Cumming’s [1] hypothesis, ovarian development was more constrained by access to mates in the ornamented *E. aestiva* females.

## 5. Ornamentation

The evolutionary origins of male-specific ornamental traits have been of interest to biologists since Darwin [56]. Among female animals generally, while sexual selection has been commonly documented, ornamental traits remain rare even when females experience strong sexual selection. When female ornaments do (rarely) evolve, it is theorised that they do so to improve attractiveness or for intrasexual competition, similar to their purpose in males [57,58]. The empidine flies represent exceptional study systems for measuring the evolutionary and ecological pressures that can shape ornaments, generally, and also how those pressures might cause sex-specific changes (ornaments) in females that do not directly mirror theoretical predictions developed to explain male ornaments [2,24,59]. 

## 6. The Evolution of Female-Specific Ornaments

As female reproductive success is typically limited more by access to resources (to improve/facilitate gamete production) than by access to mates [13,60], resource investment into costly ornamental traits might come at a higher cost to reproductive success [61]. While females might overcome costs associated with ornament expression if they receive direct benefits in the form of nuptial gifts at the time of mating [62,63], males might still prefer unornamented females if ornaments are attractive and displaying females are more likely to be polyandrous [24,47,54,61]. This negative feedback between investment in attractive signals and reproductive value is far less likely for male ornaments because male reproductive value to females is not usually constrained by trade-offs between ornament investment and gamete production. A further factor limiting male preferences for elaborate females is that males mating with the most attractive females are more likely to encounter higher risk or intensity of sperm competition [64]. The relationship between female pre-mating sexual selection and male post-mating sexual selection may explain why female-biased sexually dimorphic traits are not consistently associated with traditional measures of pre-mating sexual selection across the animal kingdom [65]. 

## 7. Ornament Types

The empidids have three ornament types, all of which are female-specific, and within each type there is impressive variation [1,66]. Across the group are pinnate leg scales (modified, sclerotised leg hairs that can occur on one or all sets of legs [2,24,46,67]); inflatable abdominal sacs (abdominal appendages that can be filled with air sucked in through the mouthparts and inflated for displaying in swarms [2,47,54,59]); and darkened and/or enlarged wings (sometimes with patterns [26,40]).

The various ornaments all appear to function to exaggerate the apparent size of a female (and perhaps especially a female’s abdomen) when perceived from a distance. In fact, the pinnate leg scales are most often observed in a careful position alongside the abdomen during swarming flight (see Figure 1). Such placement is intuitive if there is male choice in favour of more fecund females (as expected in insects [68]): females might use ornaments to exaggerate their apparent size under the pressure of choosy males bearing gifts. This hypothesis was supported by an ingenious experimental field study of dance flies: Funk and Tallamy [2] suspended photographic silhouettes of females within a natural (and highly female-biased) swarm of *R. longicauda*, and showed that larger silhouettes received much more attention from courting males.

In fact, while many ornamented empidid species display only a single ornament type (see [1,52,66]), some have multiple ornaments. The best studied example of multiple ornaments is *Rhamphomyia longicauda* (Figure 1) [2,46,47,59]. In contrast to Funk and Tallamy’s [2] original study, and in contrast to expectations for male sexually selected traits, the evidence for directional selection on female ornaments themselves (as opposed to overall female size) is mixed—in some cases sexual selection appears to be stabilising [59], while other evidence suggests directional selection [2,46,47,67]. Perhaps the answer lies in better understanding the nature of multiple ornaments (honest signals of quality vs. deception and sexual conflict), however, even within *R. longicauda* the story remains unclear for how multiple ornaments have evolved.

## 8. Multiple Ornament Evolution

Theories (primarily dealing with male-specific displays) have been developed to explain the origin of multiple ornaments including non-adaptive mate choice models (e.g., via sensory biases that drive mate choice [69]), adaptive models (e.g., honest signaling of traits in the more competitive sex [70]), and sexual conflict resulting from sexually antagonistic coevolution [71]. These theories can be extended/adapted to help explain how multiple female-specific ornaments have evolved. Within the empidine flies, there is theoretical and empirical evidence for both adaptive (honest) signaling [54,72] and sexual conflict, where female ornaments signal deceptively about their fecundity [2,24,47]. In [47], we argue that because the multiple ornaments displayed by *R. longicauda* (pinnate leg scales and inflatable abdominal sacs) do not combine to additively improve attraction for female flies, this is evidence that these ornaments are deceptive traits indicative of sexual conflict: antagonistic coevolution predicts cycles of evolutionary innovation in seductive traits (such as female ornaments) and resistance to seduction (in which the choosing sex becomes less susceptible to being seduced by the ornament [71,73]. The fact that female ornaments are differently effective is consistent with the development of resistance among choosy males, as is the remarkable evolutionary lability of female ornaments in the dance fly phylogeny [24]. Additionally, in [24] we show that males in species with ornamented females (which are more polyandrous than average) experience more post-copulatory sexual selection (as evidenced by larger relative testis size) across the empidine lineage. In recent work, Wiberg et al. [74] argue that males also evolve eye facet dimorphism to improve detection of deceptive ornamental traits in females. Collectively, these findings suggest that males are evolving costly traits (that improve their success in sperm competition or avoiding deception) in response to deceptive female ornaments in what we argue is a sexually antagonistic coevolutionary loop [71]. However, work from Wheeler et al. [59] and Browne and Gwynne [54] suggests a role for honesty in the evolution of empidine ornamental traits; consistent with theory predicting stabilising selection on female ornaments that honestly signal quality [72], Wheeler et al. [59] showed that female ornaments in *R. longicauda* are under stabilising selection, while Browne and Gwynne [54] showed positive allometric relationships between body size and ornaments suggesting that ornaments might be reliable cues of female value. Finally, Funk and Tallamy [2] first suggested abdominal sacs act as deceptive traits, however, given the positive relationship observed between abdomen size and egg number (Figure 9 in [2]), there may be scope for honesty as well (i.e., females with the largest ornaments also have more eggs). How much each of these mechanisms (honest signaling to exaggerate a trait and sexual conflict via sexually antagonistic coevolution) contributes to the evolution of female-specific multiple ornaments remains to be settled. However, further studies in the empidine flies, particularly comparative tests across species with multiple displays from different ornament “types”, are likely to help elucidate these complicated relationships. 

## 9. Costs of Mating and Swarming

One aspect of mating costs to male dance flies is the cost of carrying a mated (often ornamented) female and her nuptial prey while in flight. Although there are costs to male *Hilara* of carrying female and prey [75], male *R. longicauda* appear to experience no load-lifting costs of carrying larger ornamented mates and their nuptial gifts [76].

Turning to female ornamentation and swarming, sexual selection as an explanation for (mainly male) ornaments and armaments has typically assumed that these traits have survival costs [56,77]. However, others such as Wallace [78] and Grafen [79] have suggested that increased ornament expression in particular might correlate with *increased* survival ability (see Cronin’s history of sexual selection [80]). Although many individual studies have shown viability costs of male sexually selected traits, a meta-analysis of many studies [81] showed that increased trait expression was associated with greater survival. Even if ornaments have viability costs, males in better condition may have higher survival because good condition increases survival [82,83]. 

The unusual expression of female ornaments in empid flies provides an opportunity to study the cost of female ornaments. Following the observation of more female than male *R. longicauda* caught in sticky orb webs found near swarming areas [50], Gwynne, Bussière, and Ivy [84] tested the prediction that flying swarming females encumbered with abdominal and tibial ornaments would be more likely to be caught in sticky orb webs. In experiments involving both releasing the flies beneath framed orb webs and “netting” flying mated pairs using insect net frames with an orb web mounted on the frame, females were more likely than males to end up caught in the experimental webs. These experiments were followed by a study of viability selection imposed by two spider species on the ornaments of *R. longicauda* females [42]. Collecting *R. longicauda* prey flies from sticky orb webs of *Tetragnatha* (compared to surviving flies sampled during the same time period) showed that viability selection over two seasons favoured *larger* female abdomens, not supporting the Darwinian cost hypothesis on this trait. However, although there was no significant viability selection on leg scale ornaments per se, selection favoured females with shorter legs, suggesting that long (scale-covered) legs are selected against, perhaps because of difficulty in extricating the legs from sticky webs. Interestingly, in one of the seasons the leaf-covering cob webs of *Dictya* spiders, that snared the legs of flies as they landed, were common. Females with larger scale ornaments were less likely to be caught by this predator, which again is not predicted by the hypothesis that larger ornaments are costly.

## 10. Sexual Selection on Males

While dance flies are most famous for the aspects of their courtship that frequently bring about sexual contests among females (and the ornaments that such contests sometimes favour), males also experience diverse forms of sexual selection. In fact, heightened female sexual receptivity in dance flies (undoubtedly explained in part by the exploitation of mating as a foraging opportunity) seems to have had profound consequences on the nature of sexual selection on males. In this section, we consider how the relative intensity of different forms of selection is likely to vary according to the prevailing conditions across the sequence of episodes of reproduction (such as the many factors affecting the swarm sex ratio, see the mating swarms section above). 

## 11. Pre-Copulatory Sexual Selection on Males

For those species in which nuptial gifts are a precondition for female acceptance, sexual selection likely begins before males join the mating swarm, by favouring those that are best able to acquire (in the cause of exogenous nuptial gifts), produce (in the case of endogenous gifts), or retain a gift (in species in which nuptial gifts can be reused for multiple matings, e.g., *E. confusa*, [85], *E. livida*, and *E. tessellata* [30]). While there has been considerable work on the nature of prey taken by males, little is known on the factors affecting male gift acquisition. Any character that would facilitate hunting or subjugation of prey could be favoured, and should lead to dimorphisms. Among the dimorphic characters ascribed to certain dance flies, leading contenders include leg spines (which are probably used to restrain captured prey) and dichoptic (dorsoventrally differentiated) eyes. In recent work, Browne and Gwynne [54] demonstrated that male legs spines (but not the homologous female leg scales) of *R. longicauda* share a pattern of heightened allometry with many other sexually selected traits, including the female tibial-scale ornaments of this species (see section on female ornaments). This might indicate that males with longer leg spines are able to capture prey more effectively or quickly, which would facilitate frequent swarm attendance and provide an advantage in sexual selection. In species with endogenous gifts, the ability to produce gifts will itself be under sexual selection, as may be the tendency to deceive females with more easily obtained “sham gifts”, which have no nutritional value, but may suffice to secure a mating. The presence of nuptial gifts indicates a prior history of directional selection (leading to the convergent exaggeration of the traits in several lineages; see [86]), but the nature of selection at equilibrium is not consistently clear, especially for gifts that are not ingested (see section above on nuptial gifts). 

While nuptial gifts may in many cases enable courtship (with females apparently refusing mates who lack them), there are other features of behavioural performance in swarms that could also be under selection in males. Anecdotally, this could include the ability to perform seductive dances, to quickly locate and approach females, and to isolate them from rivals. It could also include the ability to maintain a preferred position in a mating lek at the expense of rivals (see section on mating swarms above).

Even though such behavioural aspects of performance are intuitive, they are quite difficult to study in the absence of detailed videographic analysis, which remains lacking. There have been several attempts to measure selection on male traits, but these understandably consist of more readily quantified male characters, such as those observable from captured specimens, and their nuptial gifts. The results of these studies make generalising across taxa difficult, in part because selection on males is not consistent even when considering comparable traits. As previously noted, Sadowski et al. [28] studied a (non-nutritious) balloon-carrying species, *E. snoddyi*, and showed that larger males carrying intermediately sized (inedible) balloons had the highest probability of being mated. In the prey-donating species *R. sulcata*, by contrast, LeBas and colleagues [87] found modest (and non-significant) but negative associations between mating success and both prey size and male size, revealed in both linear and correlational aspects of selection. However, male age did significantly predict pairing success in their study (older males were more successful). In *R. longicauda*, there is significant selection favouring males with shorter wings, and correlational selection favouring males with longer tibiae for their wing size [46]. Browne and Gwynne [54] conducted further morphometric analyses of males in the same species, and intriguingly demonstrate positive static allometry on male tibial spines alone among several other characters. While strong positive allometry is neither necessary nor sufficient evidence that positive selection is operating on a character, it is often associated with such selection [88]. The function of the male spines is not yet clear, but they could conceivably be associated with predation success insofar as they function to capture and retain prey; indeed, previous work has documented notable sexual differences in leg traits among other empidines [26,40]. A promising avenue to address this question would involve, for example, quantifying selection on leg traits associated with predation success independent of mating success (e.g., by measuring the leg traits for swarming males who have not yet secured a pair, and comparing to randomly captured males prior to prey acquisition).

Having paired with a female within an aerial swarm, mating in different species typically proceeds either on the wing or after alighting on a copulatory perch (see section on mating swarms above). At this stage, several further processes might affect the duration of copulation, which seems to co-vary directly with insemination success [89]. Marden’s [75] work (mentioned above) on wing loading in *Hilara* produced intriguing evidence that one key aspect of selection might involve the ability to carry nuptial gifts and partners, which might be limited, particularly for species with aerial swarms and in-flight copulations. However, as noted, these constraints are not evident in all taxa (see Murray et al.’s study of *R. longicauda* [76]). 

## 12. Post-Copulatory Sexual Selection on Males

As female empidines tend to be more sexually receptive than in many other insects (presumably because mating is often accompanied by the chance to feed on nutritious nuptial gifts), a large component of sexual selection on males occurs after copulation, a point that has been largely neglected in studies of animals showing “role reversals” in mating and courtship behaviour because the emphasis has been on pre-mating behaviour and sexual selection on females. The most obvious mediating behaviour in contests over insemination success are the nuptial gifts themselves. Insemination is very likely to occur gradually in empidines, which means that larger gifts should generally lead to longer copulations and greater sperm transfer [23,89]. Consequently, while acquiring a nuptial gift earns the male the opportunity to compete for a mating, acquiring a large and tasty gift may be needed to best convert its acquisition into substantial sperm transfer.

Little is known about other traits under selection due to sperm competition. Murray et al. [24] showed that species with elaborate ornaments tended to have males with larger testes, which is consistent with either heightened insemination of individual females (to provide an edge in numerical sperm competition), more frequent mating because of heightened receptivity, or both [90]. Variation in the reproductive morphology of females is also undoubtedly important for setting the stage in which sperm competition occurs. There are no systematic studies of reproductive tract characteristics in males and females, but such work would be a very welcome addition, particularly in light of the relatively elaborate genitalic capsules to be found among males and the curious observation of species-level variation in the sclerotisation of spermathecae (RLM, pers. obs.). 

Some recent work has attempted to assess patterns of paternity in empidines. Herridge [91], for example, showed that the males with the highest representation in spermathecal stores (judged by the amplification of microsatellite markers) were not substantially overrepresented relative to an ideal lottery (i.e., there was no strong evidence of sperm displacement by later males, or ejaculate plugs inserted by first males). Browne and Gwynne [54] provided complementary evidence by analysing the parentage of developing eggs laid by wild-captured females of *R. longicauda*. Their findings also support a fair contest among ejaculates: there is no evidence of a last-male advantage, and the proportion of offspring sired by males (regardless of whether they were the last mate or a previous mate, whose genotype was inferred on the basis of brood genetic variation) did not deviate substantially from expectations based on equal paternity. Such “reduced cuckoldry” paternity sharing (in female lifetime egg laying) is also seen in animal species with exclusive male parental care and is similar to that found in the few studies of paternity in other insects with valuable nuptial gifts [54].

## 13. Sexual Conflict

An intriguing but as yet difficult-to-quantify aspect of variation in fitness among males concerns variation in mate quality. As females in some species compete intensely for access to mates, and because males in some cases present considerable investments (in the form of nuptial gifts) that might take time and effort to reacquire or regenerate, relatively strong male choice is expected. As noted above, for most insects [68], a primary target of such male choice is expected to be female fecundity—females with more eggs could conceivably provide their mates with higher reproductive success. Such selection on males is probably best considered as natural selection, rather than sexual selection, however, since it involves male choice among rival females (imposing sexual selection on females), rather than male contests for access to the females. 

Male preference for certain females risks undermining the benefits of mating with them (see section on female ornaments above) because more popular females probably provide males with higher sperm competition risk or intensity. How such negative feedback is resolved remains unclear, and likely depends on the extent to which additional mating meals can increase fecundity (and potentially offset the loss in paternity to rival males), as well as the patterns of sperm precedence [54,91]. For example, if the last male to mate has an advantage, then males who can find highly gravid females (who are just about to lay eggs) might not suffer large costs from female promiscuity. In these cases, the timing of a female’s egg development might be more useful as a signal of reproductive value than the number of eggs she bears. We therefore might expect that selection on male empidines favours traits that lead to matings with females of high reproductive value, either because those females are more fecund, more gravid (which might require sensory cues of large size), or present low risks or intensities of sperm competition. 

Intriguingly, it may be that female ornaments evolved to signal large size deceptively (in order to improve the chances of acquiring mates and nuptial gifts) regardless of their current ovarian condition. In such cases, male traits that could distinguish deceptive signals of size from honest cues of ovarian development might be under selection. Wiberg et al. [74] provide intriguing evidence that the dichoptic eyes of male dance flies may in fact be favoured for just this reason, to see past the “disguises” that female ornaments provide by exaggerating the appearance of size regardless of ovarian development. This argument is supported by the strong association across species between male dichoptic eyes and female tibial scales, ornaments that might best be distinguished from abdominal girth if male eyes were so photosensitive that they could detect stray photons appearing between legs and abdomens during swarming dances.

Deception seems to have engendered selection not only on perceptual traits in males, but also on traits that could deceptively signal to females, e.g., on the presence or size of nuptial gift. Indeed, the silk balloons that attracted scientists to the curious mating behaviour of some dance flies [11] may well have evolved as a less-expensive or more easily acquired nuptial gift than prey items. Kessel [11] hypothesised that different levels of silk use represent different phylogenetic stages in an evolutionary sequence, but recent phylogenetic analyses [24,92] do not provide substantial support for a directional progression. Instead, there seem to have been many transitions or convergent evolutionary innovations in the expression of non-nutritious gifts. Inedible gifts are occasionally observable in species for which mating typically involves nutritious prey, and Lebas and Hockham [23] showed that the capacity to adopt inedible gift alternatives also exists in taxa for which such inedible gifts have not been observed. In one species of Japanese empid, there is striking dimorphism in forelegs [93], which could have evolved to deceive females insofar as swollen foretarsi could be mistaken for nutritious gifts [94]. It is worth noting that non-nutritious gifts may not prolong insemination as much as nutritious alternatives [23], and that females may well be disinclined to remain in copula upon discovery of male deception. For this reason, we might expect male copulatory organs that prevent female escape once copulation is initiated. Such organs seem more prevalent in *Hilara* flies than in the *Empis*–*Rhamphomyia* clade, at least insofar as (in our experience) *Hilara* pairs tend to remain in copula even after netting, whereas netting *Empis* and *Rhamphomyia* pairs almost always result in separation of the copulating pair (LFB, pers. obs.).

## 14. Conclusions

Empidine flies represent an excellent system in which to study sexual selection because of the impressive diversity of morphological and behavioural traits that have evolved to increase mating success across the group. The development of female-specific ornaments has allowed scientists to better understand constraints on the evolution of ornamental displays, generally, but also the limitations within evolutionary trait space for female sexually selected characters—unsurprisingly, ornament evolution in females often does not mirror their evolution/development in males. Additionally, the behaviour observed in the mating swarms of empidine flies, including “dancing” in lek-like mating arenas and the provisioning of nutritious and “cheating” nuptial gifts, has provided insights into classic measurements and theories of sexual selection including intrasexual mating competitions, operational sex ratios, and courtship role reversal. Finally, from studying behavioural and morphological traits in wild populations, researchers can use empidine flies to differentiate and measure pre- and post-copulatory sexual selection pressures, particularly as they relate to systems with strong sexual selection on females. 

Future work on sexual selection in empidines should aim to improve lab methods to raise empidine larvae and adults so that that the mainly observational work on dance flies can include more controlled, manipulative experiments. For example, how does experimental manipulation of pinnate leg scales affect mating behaviour and reproductive success? Empidine fly sexual selection research has been influential in the study of sexual conflict, which has been invoked to explain multiple ornament evolution in females [24]. Future work investigating the genetic architecture of male preference for, and female evolution of, deceptive signals would be an exciting potential next step in teasing apart how sexual conflict contributes to the diversity of sexually selected traits observed in empidine flies, and improve our understanding of how females can evolve costly traits that improve access to males/nuptial gifts. Given the recent phylogenies of the group that have become available, the coevolutionary dynamics across the group could be studied using rapidly evolving traits (e.g., genitalia) [95]. For example, how does strong sexual selection on females (using ornamentation as a proxy) relate to genital complexity? Finally, in the current world of online digital resources, empidine flies, which are speciose and abundant in many parts of the globe, would benefit from community science (e.g., iNaturalist, BugGuide) engagement. There are more than 76,000 observations worldwide from 495 species within the Empidoidea superfamily (as of April 2022 on iNaturalist) that, with curation, could be employed to answer questions about empid sexual selection through time and space. For example, how does the expression of ornamental traits change across species’ ranges (e.g., as a function of differences in prey availability or sex-specific mortality)? How do sexually selected traits alter where empidine flies are found across an urban–rural landscape? How do male and female empids contribute to pollination services (e.g., by recording photos of empids on flowers [96,97])? With relatively trivial amounts of training, measures of sex ratios (or even operational sex ratios) in nature could be collected for multiple populations. Making use of community science apps could allow researchers to expand their data collection to ultimately link patterns of sexual selection (e.g., relative ornament size, wing colour sexual dimorphism) to temporal and spatial patterns associated with climate change and land-use change, respectively. 

Empidinae flies represent an excellent group for studying sexual selection and mating system evolution. Many species display impressive variation in behaviour or morphology that diverges from typical patterns; this variation can provide valuable insights on the evolutionary forces that promote diversity. Through advancement in technology, animal husbandry, and community engagement, there are many exciting opportunities to learn more about sexual selection, sex-biased traits, and mating system evolution by studying Empidinae flies.

## Figures and Tables

**Table 1:** An alphabetical summary of the Empidinae species discussed in the text with associated descriptions of female ornamentation (when present) and measurements (when available) of behavioural and mating system traits.

Species	Female Ornaments Present	Nuptial Gift Type	OSR ^a^	References ^b^
*Empis aestiva*	Pinnate scales; wing colour	Prey	0.34 (0.29, 0.39)	Hunter and Bussière 2019; Murray 2017; 2020
*E. barbatoides*	none	Prey	variable from male to female biased	Alcock 1973
*E. borealis*	Enlarged and darkened wings	Prey	0.44 (0.32, 0.56)	Svensson and Petersson 1987; Svensson et al., 1989; Svensson et al., 1990
*E. confusa*	none	Prey	unknown	Chvala 1980
*E. jaschoforum*	Pinnate scales	unknown	unknown	Daugeron et al., 2011
*E. livida*	Wing colour	Prey	unknown	Preston-Mafham & Preston-Mafham 1993
*E. nigripes*	pinnate scales; wing colour	Prey	0.46 (0.30, 0.62)	Murray 2017; 2020
*E. opaca*	none	Prey; seed fluff	unknown	Preston-Mafham 1999
*E. poplitea*	none	Prey	male-biased	Alcock 1973
*E. snoddyi*	none	Inedible balloon gift	unknown	Sadowski et al., 1999
*E. tessellata*	none	Prey	0.71 (0.61, 0.81)	Murray 2017; 2020; Figure 1
*E. trigramma*	none	‘liquid gift’	unknown	Preston Mafham 1999
*Hilara* sp.	none	Prey	unknown	Marden 1989
*Rhamphomyia* sp.	Pinnate scales	Prey	unknown	Alcock 2018
*R. crassirostris*		Prey	0.34 (0.29, 0.39)	Hunter and Bussiere 2019; Murray et al., 2017; 2020
*R. dentipes*	Pinnate scales	Prey	0.73 (0.46, 0.99)	Murray et al., 2017; 2020
*R. fumosa*	Pinnate scales; abdominal sacs	Prey	female- biased	Steyskal 1941; 1942
*R. longicauda*	Pinnate scales; abdominal sacs	Prey	0.24 (0.20, 0.28)	Funk and Tallamy 2000; Gwynne and Bussiere 2002; Bussiere et al., 2008; Gwynne et al., 2007; 2015; Murray et al., 2018; 2019; 2020; Browne and Gwynne 2022; Figure 1
*R. longipes*	Pinnate scales	Prey	0.71 (0.67, 0.71)	Murray et al., 2017; 2020
*R. magellensis*	none	none	unknown	Daugeron and Grooteart 2005
*R. marginata*	Enlarged, patterned wings	Prey	0.04	Svensson 1997
*R. sulcata*	none	Prey	0.63 (0.54, 0.99)	LeBas et al., 2004; LeBas and Hockham 2005
*R. sociabilis*	Pinnate scales	Prey	0.67	Evans 1988
*R. tarsata*	Pinnate scales	Prey	unknown	LeBas et al., 2003
*R. tibiella*	Abdominal sacs	Prey	0.59 (0.46, 0.74)	Murray et al., 2017; 2020

a—OSR (Operational Sex Ratio) measured as the proportion of males (smaller values are more female-biased) collected within mating swarms. Most numerical estimates and upper and lower binomial confidence intervals are from Murray et al., 2020; exceptions include *R. marginata* from Svensson 1997, *R. sociabilis* from Evans 1988 and any descriptions of swarms with a sex bias are from the listed reference. b—We have listed references associated with each taxon mentioned in the review, but for species with many references, these lists are not always exhaustive.

## Data Availability

Not applicable.

## References

[1] Cumming J.M. (1994). Sexual selection and the evolution of dance fly mating systems (Diptera: Empididae; Empidinae). Can. Entomol..

[2] Funk D.H., Tallamy D.W. (2000). Courtship role reversal and deceptive signals in the long-tailed dance fly, *Rhamphomyia longicauda*. Anim. Behav..

[3] Darwin C. (1860). Letter to Asa Gray. https://www.darwinproject.ac.uk/letter/DCP-LETT-2743.xml.

[4] Darwin F. (1911). The Life and Letters of Charles Darwin.

[5] Thornhill R., Dodson G., Marshall L. (1983). Sexual selection and insect mating behavior. Am. Biol. Teach..

[6] Shuker D.M., Kvarnemo C. (2021). The definition of sexual selection. Behav. Ecol..

[7] Hosken D., House C. (2011). Sexual selection. Curr. Biol..

[8] Lewis S.M., Vahed K., Koene J.M., Engqvist L., Bussière L.F., Perry J.C., Gwynne D., Lehmann G.U.C. (2014). Emerging issues in the evolution of animal nuptial gifts. Biol. Lett..

[9] Downes J.A., Smith S. (1969). New or little known feeding habits in Empididae. Can. Entomol..

[10] Downes J.A. (1970). The feeding and mating behaviour of the specialized Empidinae (Diptera); Observations on four species of *Rhamphomyia* in the high arctic and a general discussion. Can. Entomol..

[11] Kessel E.L. (1955). The mating activities of balloon flies. Syst. Zool..

[12] Williams G. (1966). Adaptation and Natural Selection.

[13] Trivers R.L., Campbell B. (1972). Parental investment and sexual selection. Sexual Selection and The Descent of Man, 1871–1971.

[14] Thornhill R. (1976). Sexual selection and paternal investment in insects. Am. Nat..

[15] Thornhill R., Blum M.S., Blum N.A. (1979). Male and female sexual selection and the evolution of mating strategies in insects. Sexual Selection and Reproductive Competition in Insects.

[16] Gwynne D.T. (1981). Sexual difference theory: Mormon crickets show role reversal in mate choice. Science.

[17] Thornhill R., Gwynne D.T. (1986). The evolution of sexual differences in insects. Am. Sci..

[18] Tang C., Li X., Liu X., Engel M., Liao H., Yang D. (2022). A Cretaceous balloon lifts the veil on the antiquity and evolution of nuptial gifts. Gondwana Res..

[19] Wahlberg E., Johanson K. (2018). Molecular phylogenetics reveals novel relationships within Empidoidea (Diptera). Syst. Entomol..

[20] Laurence B. (1953). On the feeding habits of *Blinocera* (*Wiedemannia*) *bistigma* Curtis(Diptera: Empididae). Proc. R. Soc..

[21] Rogers E. (1981). Mating in *Wiedemannia* (Diptera: Empididae). J. Nat. Hist..

[22] Newkirk M.R. (1970). Biology of the longtailed dance fly, *Rhamphomyia longicauda* (Diptera: Empididae). Ann. Entomol. Soc. Am..

[23] LeBas N.R., Hockham L.R. (2005). An Invasion of cheats. Curr. Biol..

[24] Murray R.L., Herridge E.J., Ness R.W., Wiberg R., Bussière L.F. (2020). Competition for access to mates predicts female-specific ornamentation and male investment in relative testis size. Evolution.

[25] Alcock J. (1973). The mating behaviour of *Empis barbatoides* Melander and *Empis poplitea* Loew (Diptera: Empididae). J. Nat. Hist..

[26] Svensson B.G., Petersson E. (1987). Sex-role reversed courtship behavior, sexual dimorphism and nuptial gifts in the dance fly, *Empis borealis* (L). Ann. Zool. Fenn..

[27] Preston-Mafham K.P. (1999). Courtship and mating in *Empis* (*Xanthempis*) *trigramma* Meig., *E. tessellata* F. and E.(*Polyblepharis*) *opaca* F.(Diptera: Empididae) and the possible implications of ‘cheating’ behaviour. J. Zool..

[28] Sadowski J.A., Moore A.J., Brodie E.D. (1999). The evolution of empty nuptial gifts in a dance fly, *Empis snoddyi* (Diptera: Empididae): Bigger isn’t always better. Behav. Ecol. Sociobiol..

[29] Aldrich J., Turley L. (1899). A balloon-making fly. Am. Nat..

[30] Preston-Mafham R., Preston-Mafham K. (1993). The Encyclopedia of Land Invertebrate Behaviour.

[31] Hennessey R. (2018). *Hilara* sp. (Diptera: Empididae; Empidinae): Mating system, swarm movements, and inbreeding avoidance. J. Insect Behav..

[32] Daugeron C., Grootaert P. (2005). Atypical mating behaviour in the empidine dance fly *Rhamphomyia* (*Lundstroemiella*) *magellensis* (Diptera: Empididae: Empidinae). Belg. J. Zool..

[33] Melander A., Wytsman P. (1928). Diptera, Fam. Empididae. Genera Insectorum.

[34] Downes J.A. (1978). Feeding and mating in the insectivorous Ceratopogoninae (Diptera). Mem. Entomol. Soc. Can..

[35] Thornhill R. (1976). Sexual selection and nuptial feeding behavior in *Bittacus apicalis* (Insecta: Mecoptera). Am. Nat..

[36] Thornhill R., Alcock J. (1983). The Evolution of Insect Mating Systems.

[37] Evans H.E. (1988). Observations on swarms of *Rhamphomyia sociabilis* (Williston) (Diptera:Empididae). J. New York Entomol. Soc..

[38] Downes J.A. (1969). Feeding and mating in dance fly *Rhamphomyia nigrita* (Diptera-Empididae). Am. Zool..

[39] Steyskal G. (1941). A curious habit of an empidid fly. Bull. Brooklyn Entomol. Soc..

[40] Svensson B.G. (1997). Swarming behavior, sexual dimorphism, and female reproductive status in the sex role-reversed dance fly species *Rhamphomyia marginata*. J. Insect Behav..

[41] Alcock J. (2016). The mating behavior of an undescribed species of *Rhamphomyia* (Diptera: Empididae). J. Insect Behav..

[42] Gwynne D.T., Punzalan D., Hunt J. (2015). Viability selection on female fly finery in the wild. Biol. J. Linn. Soc..

[43] Olson R.O., Hintze A., Dyer F., Knoester D.B. (2013). Predator confusion is sufficient to evolve swarming behaviour. J. R. Soc. Interface.

[44] Hovi M., Alatalo R., Höglund J., Lundberg A., Rintamäki P. (1994). Lek centre attracts black grouse females. Proc. R. Soc. B-Biol. Sci..

[45] Thornhill R. (1980). Sexual selection within mating swarms of the lovebug, *Plecia nearctica* (Diptera: Bibionidae). Anim. Behav..

[46] Bussière L.F., Gwynne D.T., Brooks R. (2008). Contrasting sexual selection on males and females in a role-reversed swarming dance fly, *Rhamphomyia longicauda* Loew (Diptera: Empididae). J. Evol. Biol..

[47] Murray R.L., Wheeler J., Gwynne D.T., Bussière L.F. (2018). Sexual selection on multiple female ornaments in dance flies. Proc. R. Soc. B-Biol. Sci..

[48] Emlen S.T., Oring L.W. (1977). Ecology, sexual selection, and the evolution of mating systems. Science.

[49] Kokko H., Klug H., Jennions M.D. (2012). Unifying cornerstones of sexual selection: Operational sex ratio, Bateman gradient and the scope for competitive investment. Ecol. Lett..

[50] Gwynne D., Bussière L. (2002). Female mating swarms increase predation risk in a role-reversed dance fly (Diptera: Empididae: *Rhamphomyia longicauda* Loew). Behaviour.

[51] Jiggins F.M., Hurst G.D., Majerus M.E. (2000). Sex-ratio-distorting *Wolbachia* causes sex-role reversal in its butterfly host. Proc. R. Soc. B Biol. Sci..

[52] Murray R.L., Herridge E.J., Ness R.W., Bussière L.F. (2017). Are sex ratio distorting endosymbionts responsible for mating system variation among dance flies (Diptera: Empidinae)?. PLoS ONE.

[53] Ahnesjö I., Kvarnemo C., Merilaita S. (2001). Using potential reproductive rates to predict mating competition among individuals qualified to mate. Behav. Ecol..

[54] Browne J.H., Gwynne D.T. (2022). Deceived, but not betrayed: Static allometry suggests female ornaments in the long-tailed dance fly (*Rhamphomyia longicauda*) exaggerate condition to males. Evol. Ecol..

[55] Hunter F.D., Bussière L.F. (2019). Comparative evidence supports a role for reproductive allocation in the evolution of female ornament diversity. Ecol. Entomol..

[56] Darwin C. (1871). The Descent of Man and Selection in Relation to Sex.

[57] Amundsen T. (2000). Why are female birds ornamented?. Trends Ecol. Evol..

[58] Tobias J.A., Montgomerie R., Lyon B.E. (2012). The evolution of female ornaments and weaponry: Social selection, sexual selection and ecological competition. Philos. Trans. R. Soc. B Biol. Sci..

[59] Wheeler J., Gwynne D.T., Bussière L.F. (2012). Stabilizing sexual selection for female ornaments in a dance fly. J. Evol. Biol..

[60] Clutton-Brock T.H., Parker G.A. (1992). Potential reproductive rates and the operation of sexual selection. Q. Rev. Biol..

[61] Fitzpatrick S., Berglund A., Rosenqvist G. (1995). Ornaments or offspring: Costs to reproductive success restrict sexual selection processes. Biol. J. Linn. Soc..

[62] Vahed K. (1998). The function of nuptial feeding in insects: Review of empirical studies. Biol. Rev. Camb. Philos. Soc..

[63] South A., Lewis S.M. (2012). Determinants of reproductive success across sequential episodes of sexual selection in a firefly. Proc. R. Soc. B-Biol. Sci..

[64] Herridge E.J., Murray R.L., Gwynne D.T., Bussière L.F. (2016). Diversity in mating and parental sex roles. Encyclopedia of Evolutionary Biology.

[65] Janicke T., Morrow E.H. (2018). Operational sex ratio predicts the opportunity and direction of sexual selection across animals. Ecol. Lett..

[66] Collin J.E. (1961). British Flies VI: Empididae Part 2: Hybotinae, Empidinae (Except Hilara).

[67] LeBas N.R., Hockham L.R., Ritchie M.G. (2003). Nonlinear and correlational sexual selection on honest female ornamentation. Proc. R. Soc. B Biol. Sci..

[68] Bonduriansky R. (2001). The evolution of male mate choice in insects: A synthesis of ideas and evidence. Biol. Rev..

[69] Ryan M.J. (2018). A Taste for The Beautiful: The Evolution of Attraction.

[70] Andersson M. (1986). Evolution of condition-dependent sex ornaments and mating preferences: Sexual selection based on viability differences. Evolution.

[71] Arnqvist G., Rowe L. (2005). Sexual Conflict.

[72] Chenoweth S.F., Doughty P., Kokko H. (2006). Can non-directional male mating preferences facilitate honest female ornamentation?. Ecol. Lett..

[73] Holland B., Rice W.R. (1998). Perspective: Chase-away sexual selection: Antagonistic seduction vs. resistance. Evolution.

[74] Wiberg R., Murray R.L., Herridge E.J., Gwynne D.T., Bussière L.F. (2022). What are you lookin’ at?: A role for sexual conflict in coevolution of female display signals and male sensory adaptations?. bioRxiv.

[75] Marden J.H. (1989). Effects of load-lifting constraints on the mating system of a dance fly. Ecology.

[76] Murray R.L., Gwynne D.T., Bussière L.F. (2019). The role of functional constraints in nonrandom mating patterns for a dance fly with female ornaments. J. Evol. Biol..

[77] Fisher R. (1930). The Genetical Theory of Natural Selection.

[78] Wallace A. (1889). Darwinism: An Exposition of Natural Selection with Some of Its Applications.

[79] Grafen A. (1990). Biological signals as handicaps. J. Theor. Biol..

[80] Cronin H. (1991). The Ant and the Peacock: Altruism and Sexual Selection from Darwin to Today.

[81] Jennions M.D., Moller A.P., Petrie M. (2001). Sexually selected traits and adult survival: A meta-analysis. Q. Rev. Biol..

[82] Rowe L., Houle D. (1996). The lek paradox and the capture of genetic variance by condition dependent traits. Proc. R. Soc. B Biol. Sci..

[83] Tomkins J.L., Radwan J., Kotiaho J.S., Tregenza T. (2004). Genic capture and resolving the lek paradox. Trends Ecol. Evol..

[84] Gwynne D.T., Bussière L.F., Ivy T.M. (2007). Female ornaments hinder escape from spider webs in a role-reversed swarming dance fly. Anim. Behav..

[85] Chvála M. (1980). Swarming rituals in two *Empis* and one *Bicellaria* species (Diptera, Empididae). Acta Entomol. Bohemoslov..

[86] Turner S.P. (2012). The Evolution of Sexually Selected Traits in Dance Flies. Entomology. Ph.D. Thesis.

[87] LeBas N.R., Hockham L.R., Ritchie M.G. (2014). Sexual selection in the gift-giving dance fly, *Rhamphomyia sulcata*, favors small males carrying small gifts. Evolution.

[88] Bonduriansky R., Day T. (2003). The evolution of static allometry in sexually selected traits. Evolution.

[89] Svensson B.G., Petersson E., Frisk M. (1990). Nuptial gift size prolongs copulation duration in the dance fly *Empis borealis*. Ecol. Entomol..

[90] Vahed K. (2006). Larger ejaculate volumes are associated with a lower degree of polyandry across bushcricket taxa. Proc. Biol. Sci..

[91] Herridge E.J. (2016). The Role of Polyandry in Sexual Selection among Dance Flies. Ph.D. Thesis.

[92] Moulton J.K., Wiegmann B.M. (2007). The phylogenetic relationships of flies in the superfamily Empidoidea (Insecta: Diptera). Mol. Phylogenet. Evol..

[93] Daugeron C., Plant A., Winkler I., Stark A., Baylac M. (2011). Extreme male leg polymorphic asymmetry in a new empidine dance fly(Diptera: Empididae). Biol. Lett..

[94] Ritchie M., Vahed K. (2011). Sexual selection: Do flies lie with asymmetric legs?. Curr. Biol..

[95] Simmons L.W., Fitzpatrick J.L. (2019). Female genitalia can evolve more rapidly and divergently than male genitalia. Nat. Commun..

[96] Lefebvre V., Fontaine C., Villemant C., Daugeron C. (2014). Are Empidine dance flies major flower visitors in alpine environments? A case study in the Alps, France. Biol. Lett..

[97] Lefebvre V., Daugeron C., Villemant C., Fontaine C. (2019). Empidine dance flies pollinate the woodland geranium as effectively as bees. Biol. Lett..

